# Sugars, organic acids, and phenolic compounds of ancient grape cultivars (*Vitis vinifera* L.) from Igdir province of Eastern Turkey

**DOI:** 10.1186/0717-6287-48-2

**Published:** 2015-01-13

**Authors:** Sadiye Peral Eyduran, Meleksen Akin, Sezai Ercisli, Ecevit Eyduran, David Maghradze

**Affiliations:** Agricultural Faculty, Department of Horticulture, Igdır University, Igdir, Turkey; Agricultural Faculty, Department of Horticulture, Oregon State University, Corvallis, Oregon USA; Agricultural Faculty, Department of Horticulture, Atatürk University, Erzurum, Turkey; Agricultural Faculty, Department of Animal Science, Biometry Genetics Unit, Igdır University, Igdır, Turkey; Scientific-Reaserch Center of Agriculture, Tbilisi, Georgia

**Keywords:** Table grape, Biochemical composition, HPLC, Spectrophotometer, Germplasm characterization

## Abstract

**Background:**

The Eurasian grapevine (*Vitis vinifera* L.) is the most widely cultivated and economically important horticultural crop in the world. As a one of the origin area, Anatolia played an important role in the diversification and spread of the cultivated form *V. vinifera* ssp. *vinifera* cultivars and also the wild form *V. vinifera* ssp. *sylvestris* ecotypes. Although several biodiversity studies have been conducted with local cultivars in different regions of Anatolia, no information has been reported so far on the biochemical (organic acids, sugars, phenolic acids, vitamin C) and antioxidant diversity of local historical table *V. vinifera* cultivars grown in Igdir province. In this work, we studied these traits in nine local table grape cultivars viz. ‘Beyaz Kismis’ (synonym name of Sultanina or Thompson seedless), ‘Askeri’, ‘El Hakki’, ‘Kirmizi Kismis’, ‘Inek Emcegi’, ‘Hacabas’, ‘Kerim Gandi’, ‘Yazen Dayi’, and ‘Miskali’ spread in the Igdir province of Eastern part of Turkey.

**Results:**

Variability of all studied parameters is strongly influenced by cultivars (P < 0.01). Among the cultivars investigated, ‘Miskali’ showed the highest citric acid content (0.959 g/l) while ‘Kirmizi Kismis’ produced predominant contents in tartaric acid (12.71 g/l). The highest glucose (16.47 g/100 g) and fructose (15.55 g/100 g) contents were provided with ‘Beyaz Kismis’. ‘Kirmizi Kismis’ cultivar had also the highest quercetin (0.55 mg/l), o-coumaric acid (1.90 mg/l), and caffeic acid (2.73 mg/l) content. The highest ferulic acid (0.94 mg/l), and syringic acid (2.00 mg/l) contents were observed with ‘Beyaz Kismis’ cultivar. The highest antioxidant capacity was obtained as 9.09 μmol TE g^-1^ from ‘Inek Emcegi’ in TEAC (Trolox equivalent Antioxidant Capacity) assay. ‘Hacabas’ cultivar had the highest vitamin C content of 35.74 mg/100 g.

**Conclusions:**

Present results illustrated that the historical table grape cultivars grown in Igdir province of Eastern part of Turkey contained diverse and valuable sugars, organic acids, phenolic acids, Vitamin C values and demonstrated important antioxidant capacity for human health benefits. Further preservation and use of this gene pool will be helpful to avoid genetic erosion and to promote continued agriculture in the region.

## Background

Overwhelming evidence from *in vitro, in vivo*, epidemiological, and clinical trial data indicates that there are components in a plant-based diet, other than traditional nutrients, that can reduce cancer risk. More than a dozen classes of these biologically active plant chemicals, now known as ‘phytochemicals’ , have been identified. The majority of naturally occurring health-enhancing substances appear to be of horticultural plant origin [1–5].

The grape, cultivated since ancient times is one of the most significant edible and processing for wine crop due to its beneficial influences on human health and economic significance on a large scale [6], and Turkey is one of the major players in this market: according to FAO records, Turkey placed 6th position in the World in terms of amount of grape production according to the data of 2012 [7].

There are vast germplasm resources available within the genus *V. vinifera* throughout grape growing areas in the world. Despite the existence of thousands of cultivars of *V. vinifera* in grape producer countries, only a few dozen cultivars account for the vast majority of world-wide production. Numerous local cultivars have regional importance and historically played a significant role in many viticulture regions [6]. In fact local cultivars show great variability in particular given biochemical traits and they can be use locally for wine, raisins, and fresh market (table grapes) [6, 8, 9]. Therefore it is crucial to determine how much variability can be found in local grape cultivars for the given biochemical traits to use them in breeding activities. This may also help the growers to pick the right cultivar according to their market opportunities.

Grape berry ‘quality’ is one of the cardinal variables that determine wine quality. Berry ‘quality’ , however, is a generic term that refers to levels of a diverse range of berry chemical constituents including organic acids, sugars, phenolics, acidity etc. [9].

The presence of adequate levels of organic acids in the grape berries is one of the key factors to determine the quality of berries and wines. Conde et al. [8] underlined that in the general sense, organic acid contents of grape berries was an indicative in taste based on acid-sugar balance. Organic acids have favorable impacts on human metabolism as well [6]. The characterization of the phenolic compounds in grapes is of great importance in terms of positive contribution to human health and the organoleptic features of grape and wines. Polyphenols obtained from the grapes are reported to prevent cancer, cardiovascular, type-2 diabetes mellitus diseases and so forth [10].

Several variables viz. total soluble sugar, titratable acidity, nitrogen and phenolic compounds balance provide a major contribution in the description of the grape quality [11], which are very significant in case of table grape. Therefore, due to beneficial effects of the grape on human health, the researchers have more focused in particular on the identification of biochemical composition and bioactive compounds for grape cultivars grown in distinct geographic regions throughout the World. To exemplify, Topalovic et al. [12] evaluated the changes in sugar, organic acids, and phenolics at different dates (9, 16, 23, and 30 July) of the Cardinal table grape cultivar during ripening. Nile et al. [10] determined polyphenolic contents and antioxidant characteristics of different cultivars belonging to *Vitis vinifera*, *Vitis labrusca*, and *Vitis* hybrid. More comprehensively, Orak [13] investigated total antioxidant activities, phenolics, anthocyanins, and polyphenoloxidase activities for selected red grape cultivars and emphasized strong correlations among antioxidant capacity, total phenols, and anthocyanins. With the previous report by Sabir et al. [14], variation of several sugars, acids, and total phenols for juice of five grapevine (*Vitis spp.*) cultivars, depending on the various stages of the grape berry development, were scrutinized.

Considering all those former studies together, it is indispensible to conduct further studies on the identification of biochemical composition and phenolic contents that can influence the quality of the grape and its products, and notably, on the determination of the major factors (cultivar, year, location, harvest time and so on) linked to those contents under different climate conditions.

In Turkey, the native table grape cultivars are extensively cultivated in order of local usage fresh grape, raisin and grape juice. Therefore, the cultivation of some varieties has been recognized after the observations of plant and phenological characteristics in the arable soil of Igdır province located in the Eastern Anatolia region of Turkey. Igdir province includes well-known Agri Dagi - the same Ararat Mountain - that is recognized as a location where the Hoah’s Ark stopped and the birthplace of viticulture, when Noah after the Flooding planted vineyard and made the wine according to Bible. In spite of the examination, the detection of organic acid, phenolic compounds, and antioxidant compositions of the regional grape cultivars from Igdir province have not been yet documented which may gain more significance in improving the grape industry of the province in the future. Hence, an attempt was made in the current investigation to identify the sugars, organic acids and phenolic compounds for the local nine grape cultivars from Igdır province. The region cultivates only those local grape 2cultivars and there are no well-known international cultivars in the region – in opposite: we have to take in consideration, that the local cultivar ‘Sultanina’ of Turkish origin is widely spread in America, Australia, Mediterranean, Northern Africa, Europe and other countries [15]; as well as the cultivar ‘Askeri’ is also spread in Eastern countries like Azerbaijan, Armenia, Iran and Middle Asia [16].

## Results and discussion

### Organic acids

Samples of nine native grape cultivars available in the single collection vineyard together in Necefali village of Igdır province have been evaluated to detect organic acids including citric, tartaric, malic, succinic, and fumaric acids at the harvest time. The statistical analysis results for organic acids are summarized in Table 1. The cultivars significantly affected levels of citric, tartaric, malic, succinic, and fumaric acids (P < 0.01), implying that the cultivars was a very significant source of the variation on the organic acids. Our finding is in agreement with those reported by previous authors [14, 17, 18]. In the statistical assessment of Table 1, it was determined that coefficients of variation (%) of the organic acids ranged from 1.09 to 14.33.

**Table 1:** **Content of organic acids in the table grape cultivars from Igdir province of Turkey**

Cultivars	Citric acid g/l	Tartaric acid g/l	Malic acid g/l	Succinic acid g/l	Fumaric acid g/l
Askeri	0.642 ± 0.02b	4.71 ± 0.29f	2.27 ± 0.18cd	1.01 ± 0.06ab	0.0026 ± 0.0002b
Beyaz Kismis	0.263 ± 0.02f	4.92 ± 0.24ef	1.82 ± 0.10cde	0.34 ± 0.03de	0.0026 ± 0.0002b
El hakki	0.488 ± 0.05c	5.53 ± 0.28e	1.92 ± 0.13cd	0.57 + 0.01de	0.0012 ± 0.0001c
Hacabas	0.637 ± 0.01b	7.77 ± 0.12c	2.23 ± 0.21cd	1.34 ± 0.15a	0.0024 ± 0.0002b
Inek Emcegi	0.323 ± 0.03e	4.74 ± 0.12ef	3.29 ± 0.12ab	1.17 ± 0.10ab	0.0012 ± 0.0001c
Kerim Gandi	0.427 ± 0.02d	6.21 ± 0.09d	1.31 ± 0.16e	1.07 ± 0.03ab	0.0027 ± 0.0002b
Kırmızı Kismis	0.264 ± 0.02f	12.71 ± 0.12a	1.53 ± 0.16de	0.93 ± 0.01bc	0.0007 ± 0.0000c
Miskali	0.959 ± 0.04a	5.67 ± 0.09de	2.58 ± 0.16bc	0.25 ± 0.02e	0.0026 ± 0.0002b
Yazen Dayi	0.254 ± 0.03f	8.72 ± 0.13b	3.56 ± 0.29a	0.67 ± 0.01cd	0.0034 ± 0.0002a
CV (%)	1.09	4.90	13.44	14.33	12.38

**P < 0.01 (the significant cultivar effect) ^a,b,c,d.f^. In each column means followed by different letter are statistically different each other at P < 0.01 (**), ND: Non determined.

In citric acid, the averages of the evaluated cultivars had a great range between 0.254 to 0.959 g/l. Among the available cultivars, ‘Miskali’ produced the highest average in citric acid (0.959 g/l) and was found significantly different from the others, statistically (P < 0.01).

A very desirable coefficient of variation with 1.09 (%) was found for citric acid (Table 1).

It could be inferred from the statistical results in Table 1 that tartaric acid varied from 9.43 to 25.43 g/l in the study. As seen from Table 1, it was understood markedly that the highest tartaric acid content was averagely obtained from ‘Kırmızı Kismis’ among the assessed cultivars. Additionally, it is apparent that ‘Kırmızı Kismis’ differed from other grape cultivars, statistically.

In relation to the results reported in the Table 1, the statistically significant influence of the cultivars on content of malic acid was determined (P < 0.01). Of those local grape cultivars, ‘Yazen Dayi’ recorded the highest average content for malic acid and was equal to 3.56 mg/l. Table 1 denotes that the very narrow range of 1.31 to 3.56 g/l was decided for malic acid.

When Table 1 was statistically assessed, the succinic acid significantly influenced by cultivar factor with a very narrow range changed between 0.25 and 1.34 g/l in the investigation. The highest average was numerically recorded in ‘Hacabas’ cultivar, whereas no differences between the ‘Hacabas’ and some other cultivars (‘Kerim Gandi’ , ‘Inek Emcegi’ , and ‘Askeri’) were detected.

The very narrow range described for fumaric acid was taken notice from Table 1. ‘Yazen Dayi’ was realized to have a statistically higher content in the organic acid compared with other varieties (Table 1). However, it was displayed that ‘El Hakki’ , ‘Kırmızı Kismis’ , and ‘Inek Emcegi’ cultivars were not different in the acid content from each other, depending upon the results of Tukey test. For the ‘Cardinal’ grape variety during ripening, Topalovic et al. [12] informed to be 1.90 g/kg for tartaric acid content and 0.97 g/kg for malic acid content, respectively. For the Alphonse Lavellee, Muscat of Hamburg, Isabella, Italia, and Muscat of Alexandria grape cultivars, Sabir et al. [14] reported tartaric acid (3.8, 4.2, 5.2, 4.8, and 5.0), malic acid (3.6, 2.8, 3.4, 3.1, and 3.0 g/l), and citric acid contents (0.4, 0.3, 0.3, 0.2, and 0.3 g/l), respectively. In our examination (Table 1), malic acid contents of the grape cultivars were found to be considerably lower compared with their tartaric acid contents, which was in agreement with those of Sabir et al. [14].

### Antioxidant capacity, sugars, vitamin C

The results and statistical evaluations for the Trolox Equivalent Antioxidant Capacity (TEAC), vitamin C (ascorbic acid), and sugars (fructose and glucose) described in the present investigation are presented in the Table 2. As recognized in TEAC, vitamin C, fructose, and glucose produced the significantly wide variation due to the cultivar factor (P < 0.01). The coefficients of variation for TEAC, vitamin C, fructose, and glucose were very low, meaning that our investigation was reliable. For TEAC, with the range of 3.60 to 9.09 μmol TE g^-1^, the highest average value was achieved for ‘Inek emcegi’ cultivar, which was ascertained to be significantly different from others (P < 0.01).

**Table 2:** **Results for TEAC, vitamin C, fructose, and glucose in the table grape varieties from Igdir province of Turkey**

Cultivars	TEAC (μmol TE g ^-1^)	Vitamin C mg/100 g	Fructose g/100 g	Glucose g/100 g
Askeri	4.50 ± 0.19c	21.08 ± 0.44d	11.40 ± 0.14cd	13.88 ± 0.23c
Beyaz Kismis	4.07 ± 0.05c	30.61 ± 0.23c	15.55 ± 0.40a	16.47 ± 0.21a
El hakki	6.50 ± 0.25b	15.85 ± 0.32f	12.13 ± 0.09c	13.34 ± 0.07c
Hacabas	4.10 ± 0.20c	35.74 ± 0.33a	13.47 ± 0.22b	15.21 ± 0.08b
Inek Emcegi	9.09 ± 0.50a	11.21 ± 0.10 g	8.22 ± 0.08f	10.32 ± 0.18ef
Kerim Gandi	4.63 ± 0.17c	18.71 ± 0.49e	9.76 ± 0.07e	12.25 ± 0.16d
Kırmızı Kismis	3.65 ± 0.01c	33.55 ± 0.28b	9.33 ± 0.16e	10.49 ± 0.13e
Miskali	6.10 ± 0.15b	29.28 ± 0.20c	8.03 ± 0.37f	9.51 ± 0.27f
Yazen Dayi	3.60 ± 0.21c	15.73 ± 0.44f	10.32 ± 0.18de	11.39 ± 0.19d
CV (%)	7.71	2.50	3.50	2.50

**P < 0.01 (the significant cultivar effect) ^a,b,c,d,e,f^. In each column means followed by different letter are statistically different each other at P < 0.01 (**), ND: Non determined.

In the study, vitamin C varied between 11.21 and 35.74 mg per 100 g (Table 2). The results showed explicitly that the highest content identified on average for vitamin C as an indicator of the dietary of the foods was provided with ‘Hacabas’ cultivar, statistically (P < 0.01). On the other hand, ‘El Hakki’ and ‘Yazen Dayi’ cultivars were statistically similar and gave the lowest content for vitamin C (Table 2).

It was noted earlier by Sabir et al. [14] that the maturity degree of the grapes was strongly connected with sugar concentration, and principally was dependent upon quality criteria like sugar accumulation, phenolic compounds, and the ratio of sugar to acid. Rusjan and Korosec-Koruza [17] reported that sugar content of the grape was very significant to decide technological maturity and harvest time. Herewith, when fructose was taken into consideration in Table 2, the highest fructose average was appeared with ‘Beyaz Kismis’ cultivar, numerically and statistically (P < 0.01). The fructose gave the range of 8.03 to 15.55. The highest accumulation of glucose was defined in ‘Beyaz Kismis’ with the significant content of 16.47 g/100 g. Sabir et al. [14] reported glucose and fructose content between 86.4-107.0 g/l and 80.4-94.1 g/l, respectively.

As to Table 2, the accumulation of fructose contents for the present grape cultivars were found very similar to that of glucose contents with the verification of the very strong correlation of 0.957 (data not shown). The finding was consistent with those reported by some authors [12, 13] previously. For 15 red wine cultivars, Rusjan and Korosec-Koruza [17] obtained the range of 3.0-16.8 g/l for sucrose content, the range of 50.9- 89.9 g/l for glucose content, and for fructose content, the range of 54.8-83.9 g/l respectively. However, the total sugar accumulation ability in wine grapes is higher than of table grapes and it is depending also in place of cultivation.

### Phenolic acids

Results of statistical evaluations for several phenolic compounds of the grape cultivars under the investigation are summarized in Tables 3 and 4, respectively. Cultivar (P < 0.01) had a significantly remarkable impact on phenolic compounds, which are very important in grape juice and wine processing industry [18]. For analysis of variance for only one factor, cultivar, coefficients of variation relevant to all the compounds identified in the grape were found to have a range of 0.46 to 12.4% as illustrated in Tables 3 and 4. As appeared obviously from Tables 3 and 4, ferrulic acid of the ‘Yazen Dayi’ cultivar, and vanillic acids of ‘Hacabas’ and ‘Miskali’ cultivars among the grape cultivars analyzed in the present investigation were unidentified.

**Table 3:** **Results for phenolic acids (Part 1) in the table grape cultivars from Igdir province of Turkey**

Cultivars	Catechin (mg/l)	Rutin (mg/l)	Quercetin (mg/l)	Chlorogenic acid (mg/l)	Ferulic acid (mg/l)
Askeri	0.95 ± 0.01cd	1.14 ± 0.07e	0.31 ± 0.01d	1.28 ± 0.03e	0.11 ± 0.00d
Beyaz Kismis	1.26 ± 0.03b	2.22 ± 0.05c	0.36 ± 0.01cd	3.31 ± 0.04a	0.94 ± 0.01a
El Hakki	1.14 ± 0.04bc	2.25 ± 0.06c	0.43 ± 0.01b	2.36 ± 0.04c	0.08 ± 0.01d
Hacabas	0.95 ± 0.01cd	3.34 ± 0.02a	0.09 ± 0.00e	1.13 ± 0.02e	0.24 ± 0.01c
Inek Emcegi	0.76 ± 0.01d	2.33 ± 0.03c	0.14 ± 0.01e	3.48 ± 0.04a	0.22 ± 0.01c
Kerim Gandi	0.73 ± 0.02d	1.50 ± 0.04d	0.40 ± 0.02bc	1.22 ± 0.03e	0.12 ± 0.01d
Kırmızı Kismis	0.43 ± 0.01e	1.09 ± 0.03e	0.55 ± 0.01a	2.08 ± 0.03d	0.54 ± 0.01b
Miskali	1.26 ± 0.06b	1.18 ± 0.02e	0.17 ± 0.01d	2.80 ± 0.06b	0.23 ± 0.01c
Yazen Dayi	1.82 ± 0.13a	3.10 ± 0.02b	0.32 ± 0.01d	2.06 ± 0.03d	ND
CV (%)	0.86	3.57	6.16	3.03	4.93

**P < 0.01 (the significant cultivar effect) ^a,b,c,d,e^. In each column means followed by different letter are statistically different each other at P < 0.01 (**), ND: Non determined.

**Table 4:** **Results for phenolic acids (Part 2) in the table grape cultivars from Igdir province of Turkey**

Cultivars	o-coumaric acid (mg/l)	p-coumaric acid (mg/l)	Caffeic acid (mg/l)	Syringic acid (mg/l)	Vanillic acid (mg/l)	Gallic acid (mg/l)
Askeri	1.69 ± 0.02b	0.06 ± 0.006cd	2.24 ± 0.05b	0.86 ± 0.02c	0.45 ± 0.01a	0.83 ± 0.06b
Beyaz Kismis	0.91 ± 0.05d	0.04 ± 0.003d	1.35 ± 0.06d	2.00 ± 0.03a	0.27 ± 0.01b	1.18 ± 0.03a
El Hakki	0.96 ± 0.01d	0.18 ± 0.006a	1.77 ± 0.04c	0.27 ± 0.02e	0.24 ± 0.02bc	0.27 ± 0.01c
Hacabas	1.38 ± 0.02c	0.19 ± 0.009a	0.65 ± 0.02e	0.31 ± 0.03de	ND	0.15 ± 0.02c
Inek Emcegi	0.67 ± 0.01e	0.13 ± 0.006b	1.15 ± 0.04d	0.50 ± 0.03d	0.06 ± 0.00d	1.27 ± 0.10a
Kerim Gandi	0.34 ± 0.02f	0.07 ± 0.003c	1.62 ± 0.05c	1.20 ± 0.09b	0.23 ± 0.01c	0.10 ± 0.01c
Kırmızı Kismis	1.90 ± 0.04a	0.01 ± 0.000e	2.73 ± 0.04a	0.16 ± 0.01e	0.02 ± 0.00e	0.67 ± 0.05b
Miskali	1.05 ± 0.04d	0.06 ± 0.003 cd	2.45 ± 0.08b	0.27 ± 0.05e	ND	1.27 ± 0.06a
Yazen Dayi	0.74 ± 0.02e	0.13 ± 0.010b	0.40 ± 0.04f	1.10 ± 0.07b	0.05 ± 0.00de	0.32 ± 0.03c
CV (%)	0.46	10.10	5.23	10.50	7.48	12.40

**P < 0.01 (the significant cultivar effect) ^a,b,c,d,e,f^. In each column means followed by different letter are statistically different each other at P < 0.01 (**), ND: Non determined.

As antioxidants, plant flavonoids are the most important in free radical scavenging. In a previous review on nutritional benefits of flavonoids, it was reported that catechin among those flavonoids had the highest antioxidant activity [19]. Among the grape cultivars, ‘Yazen Dayi’ contained predominant level of 1.82 mg/l in catechin, a polyphenolic antioxidant plant metabolite, which plays a major role in microbial defense of the grape berry, when compared with the catechin levels of the others (Table 3). The cultivar including the lowest catechin content was recorded as ‘Kırmızı Kismis’. In a previous study, Breksa et al. [20] reported that the catechin contents identified for 16 raisin grape (*Vitis vinifera* L.) cultivars and selections had a very wide range of 1.8 to 209.1 μg/g. The present results demonstrated that the rutin content of the grape cultivars had a narrow range of 1.09 to 3.34 mg/l. The cultivar of the highest rutin content among the analyzed grape cultivars was verified to be ‘Hacabas’, numerically and statistically (Table 3), but the lowest content in rutin was obtained for the cultivars shown with letter “e” according to the results of Tukey test in Table 3. Breksa et al. [20] mentioned that the rutin contents with a very narrow range of 0.8 to 3.7 μg/g for 14 raisin grape (*Vitis vinifera* L.) cultivars were detected.

As to the statistical evaluation performed in quercetin, ‘Kırmızı Kismis’ was the grape cultivar that contained the utmost content in comparison to others (P < 0.01), whereas the lowest contents were established for the cultivars with letter “e”, on the basis of Tukey test, as also depicted from Table 3. The maximal chlorogenic acid content was procured with ‘Beyaz Kismis’ and ‘Inek Emcegi’ cultivars, which were statistically found similar. Obtained from Tukey test, the letter “e” symbolized the lowest chlorogenic acid content (Table 3). In Ferulic acid, ‘Beyaz Kismis’ cultivar was superior to the others, statistically (P < 0.01), and the cultivars shown through the letter “d” produced the lowest contents (Table 3).

Of the probed local grapes, ‘Kırmızı Kismis’ had much more content in o-coumaric acid than the others, but ‘Kerim Gandi’ with the letter “f” produced much lower content as compared to the other cultivars as also understood from Table 4. ‘Kirmizi Kismis’ was found as the cultivars with the leading average caffeic acid content of 2.73 mg/l (Table 4). In terms of syringic acid, the highest content was proved with ‘Beyaz Kismis’ cultivar, which was different from the others, statistically (P < 0.01). With Table 4, it was obvious that, ‘Askeri’ was the cultivar with the most vanillic acid content when compared with the other cultivars. Amongst the cultivars under study, ‘Beyaz Kismis’ , ‘Inek Emcegi’ , and ‘Miskali’ , statistically similar to each other, were determined to have the highest contents of gallic acid in reference to the results of Tukey test. Breksa et al. [20] reported that the gallic acid contents of only two genotypes (A95-15 and A95-27) among 16 raisin grape (*Vitis vinifera* L.) cultivars and selections were identified as 6.9 and 24.5 μg/g, respectively. In terms of the grape characterization, analyzing phenolic and aromatic compounds which can be varied to cultivar and *Terroir* of the grape is necessary for producing very high qualified wines.

## Conclusions

The obtained results reflected that the historical nine table grape cultivars grown in the Igdir province of Eastern Turkey contained valuable sugars, organic acids, and phenolic compounds in health benefits. In the development of the grape industry of Igdır province, the present study will provide industrialists at first and then breeders to get baseline information for selection of grape cultivars for cultivation or breeding programs, which may be gained great importance with the support of the further studies to be carried out more comprehensively in future years.

## Methods

### Plant material

The present investigation was conducted on nine historical local table grape cultivars ‘Beyaz Kismis’ , ‘Askeri’ , ‘El Hakki’ , ‘Kirmizi Kismis’ , ‘Inek Emcegi’ , ‘Hacabas’ , ‘Kerim Gandi’ , ‘Yazen Dayi’ , and ‘Miskali’ grown in a single collection vineyard of Igdir province - located in the Eastern Anatolia Region of Turkey. The aim is to identify their biochemical content (sugars, organic acids, and phenolic acids, Vitamin C) and antioxidant capacity. In fact the prime name of ‘Beyaz Kismis’ is ‘Sultanina’ or Thompson seedless [21]. ‘Hacabas’ and ‘Kirmizi Kismis = Kismis Kirmizi’ are also mentioned in Armenia as a country of origin (19). ‘Yazen Dayi’ - No one variety with this name is mentioned yet neither in VIVC [21] nor in European Vitis Database [22]. Some basic characteristics of these cultivars are shown in Table 5.

**Table 5:** **Basic morphological characteristics of studied cultivars**

Cultivars	Bunch weight (g)	Bunch size	Berry color	Berry shape	pH	SSC (%)	Usage
Askeri	157	High	Light Yellow	Spherical	4.50	20.5	Fresh
Beyaz Kismis	129	Medium	Light Yellow	Spherical	3.34	22.5	Fresh
El Hakki	266	Medium	Red	Spherical	3.75	16.7	Fresh
Hacabas	338	High	Light Yellow	Spherical	3.71	18.3	Fresh
Inek Emcegi	431	High	White	Elipsoidal elongated	4.03	18.3	Fresh
Kerim Gandi	147	Medium	Light Green	Elipsoidal elongated	3.80	21.0	Fresh
Kırmızı Kismis	417	High	Pink Red	Spherical	3.54	22.4	Fresh
Miskali	515	High	Light Pink	Spherical	4.08	19.0	Fresh
Yezan Dayi	269	High	Light Yellow	Spherical	3.61	25.1	Fresh

The collection site is located in the village of Necefali (40**°**10′N latitude, 44**°**4′E longitude on the 865 meters above sea level) at a distance of 13 km to the city Igdir - the administrative center of the province.

Fruits (berries) for analyses were collected from bunch of 8 grapevine plants ensured good representative of the cultivars to make average sampling, and the berries of those grape cultivars were taken during commercial ripe stage of the grapes in the dates varying between 1 to 20 August of the year 2013. Thus the sampling structured according to differential ripening dates across cultivars.

The plants were established with planting scheme of 2.5 m × 2.5 m in distance. In winter months, plants were covered by soil to avoid winter injury. Pruning is applying in February and 3–4 buds left on shoots.

### Extraction and detection of organic acids

Samples were stored at -20°C until processed. In the present paper succinic, citric, malic, fumaric and tartaric acids as organic acids were determined. Organic acids were extracted after the method suggested by Bevilacqua and Califano [23] was modified. In the study, 5 g from the whole fruit samples were transferred to centrifuge tubes. 20 ml of 0.009 N H_2_SO_4_ was pured on the samples and homogenized (Heidolph Silent Crusher M, Germany). Then, the samples were mixed for 1 hour on a shaker (Heidolph Unimax 1010, Germany) and centrifuged at 15000 g for 15 min. Their watery part extracted from the centrifuge was initially distilled from filter paper and then distilled twice from a membrane filter of 0.45 μm, and lastly, it was distilled from the cartridge SEP-PAK C_18_. Organic acids were exposed to a HPLC device on the basis of the method identified by Bevilacqua and Califano [23].

In the HPLC system, Aminex HPX - 87 H, 300 mm × 7.8 mm column (Bio-Rad Laboratories, Richmond, CA, ABD), was used and the HPLC device was commanded by Agilent package program. In the system, DAD detector (Agilent, USA) was adjusted to the wavelengths of 214 and 280 nm.

### Extraction and detection of phenolic acids

In the present paper, gallic acid, catechin, chlorogenic acid, caffeic acid, p-cumaric acid, o-cumaric acid, ferulic acid, vanillic acid, rutin, syringic acid and quercetin as phenolic acids were identified. The phenolic compounds were identified with HPLC using the modified method of Rodriguez-Delgado et al. [24].

The samples were mixed with distilled water in a ratio of 1:1, and centrifuged for 15 min at 15000 rpm. Afterwards, the upper part was filtered with 0.45 μm millipore filters, and enjected to HPLC. Chromatographic analysis was performed by Agilent 1100 (Agilent) HPLC system using DAD dedector (Agilent. USA) and 250*4.6 mm, 4 μm ODS column (HiChrom, USA). As a mobile phase, A methanol:acetic acid:water (10:2:28) and B methanol:acetic acid.water (90:2:8) were used. The extraction was made at 254 and 280 nm, 1 mL/min flow rate, and 20 μL injection volume.

### Extraction and detection of sugars

The analysis of sugars was performed using the modified method of Melgarejo et al. [25]. The sugar analyses were done with the standards of fructose and glucose of fruit juice. The sample of 5 g was homogenized and centrifuged at 12000 rpm for 2 min, afterwhich run in SEP-PAK C18 column. The extraction was preserved at -20°C until analysis. The sugars from the samples to be filtered were determined using μbondapak-NH_2_ column with 85% acetonitrile liquid phase in HPLC that has a refractive index detector. Calculation of the concentrations was done based on fruit juice standards.

### Analysis of vitamin C (Ascorbic Acid)

The sample of 5 g from the whole fruits was transferred to test tubes and then 5 ml 2.5% M-phosphoric acid solution was poured on it. The mixture was centrifuged with 6500 g for 10 min at 4°C. From the clear part in the centrifuge tube 0.5 ml was taken and 2.5% M-phosphoric solution was poured until reaching 10 ml. The new mixture was filtered by 0.45 μm teflon filter and injected to HPLC. Ascorbic Acid was detected by the C18 column (Phenomenex Luna C18, 250 × 4.60 mm, 5 μ) in the HPLC. Ultra distilled water was used as a mobile phase with 1 ml/min flow rate and pH of 2.2 adjusted with H_2_SO_4_. The DAD detector with 254 nm wavelength was used for the readings. For determination of ascorbic acid different concentration levels of L-ascorbic acid (SigmaA5960) (50, 100, 500, 1000, and 2000 ppm) were used [26].

### Detection of trolox equivalent antioxidant capacity (TEAC)

For a standard TEAC measurement, ABTS acetate was solved in a buffer and potassium persulphate was prepared [27]. To keep the stability of the mixture for a long time, 20 mM sodium acetate buffer solution was solved in an acidic pH of 4.5 and an absorbance of 734 nm, 0.700 ± 0.01. For spectrophotometric measurements, 3 ml ABTS^+^ solution was mixed with 20 μl fruit extract, and incubated for 10 min. Absorbance values obtained for 734 nm.

### Statistical analysis

Eight grapevine plants for each cultivar were used for sampling. Fruits (berries) were collected from different bunch in these 8 plants per cultivar. The descriptive statistics for all quantitative characteristics under investigation were expressed as Mean ± SE. The data were analyzed through One-Way ANOVA with four replications and mean separation was done by using Tukey multiple comparison test. In the present paper, a significance level of 1% was considered. All statistical computations were performed using SPSS 20 program.
